# Pre-Treatment of Furniture Waste for Smokeless Charcoal Production

**DOI:** 10.3390/ma13143188

**Published:** 2020-07-17

**Authors:** Paweł Kazimierski, Paulina Hercel, Katarzyna Januszewicz, Dariusz Kardaś

**Affiliations:** 1 Institute of Fluid Flow Machinery, Polish Academy of Sciences, Fiszera 14, 80-231 Gdańsk, Poland; pkazimierski@imp.gda.pl (P.K.); phercel@imp.gda.pl (P.H.); dk@imp.gda.pl (D.K.); 2 Department of Energy Conversion and Storage, Chemical Faculty, Gdańsk University of Technology, Narutowicza 11/12, 80-233 Gdańsk, Poland

**Keywords:** smokeless fuel, smokeless coal, pyrolysis, furniture waste

## Abstract

The aim of this study was to assess the possibility of using furniture waste for smokeless fuel production using the pyrolysis process. Four types of wood-based wastes were used in the pyrolysis process: pine sawdust (PS), chipboard (CB), medium-density fiberboard (MDF), and oriented strand board (OSB). Additionally, the slow and fast types of pyrolysis were compared, where the heating rates were 15 °C/min and 100 °C/min, respectively. Chemical analyses of the raw materials and the pyrolysis product yields are presented. A significant calorific value rise was observed for the solid pyrolysis products (from approximately 17.5 MJ/kg for raw materials up to approximately 29 MJ/kg for slow pyrolysis products and 31 MJ/kg for fast pyrolysis products). A higher carbon content of char was observed in raw materials (from approximately 48% for raw materials up to approximately 75% for slow pyrolysis products and approximately 82% for fast pyrolysis products) than after the pyrolysis process. This work presents the possibility of utilizing waste furniture material that is mostly composed of wood, but is not commonly used as a substrate for conversion into low-emission fuel. The results prove that the proposed solution produced char characterized by the appropriate properties to be classified as smokeless coal.

## 1. Introduction

The emission of CO_2_, one of the greenhouse gases, from coal combustion for electricity and heat generation in 2017 amounted to 98 billion tons worldwide and 1.1 billion tons in the EU [1]. It was estimated that air pollutant emissions worldwide amounted to 11.4 million tons of SO_x_, 25 million tons of NO_x_, and 79 million tons of CO in 2017 [2]. Other controlled air pollutants involved with the combustion of fossil fuels are PM10 and PM 2.5, for which the total amount in 2017 in the EU was estimated at 2.6 million tons of PM10 and 1.6 million tons of PM2.5 [2]. Air pollution is a global problem in many regions, especially those with a highly developed industrial sector. Pollution is also caused by the transportation sector and incomplete combustion in local small-scale installations used during the heating season. Using smokeless coal instead of traditional (with high volatile content) fuel could be one of the solutions that improves the problem of air pollution.

Coke is a fuel that is almost free from volatiles when combusted. An alternative for traditional fossil fuels dedicated to low-power furnaces and heaters could be a smokeless coal/char. The advantage of this material is the amount of volatiles produced during the process of combustion, which is lower than in traditional fuels. Additionally, lower air pollutants are generated by their completed combustion process.

Smokeless char is a thermally processed fuel that is partly devolatilized but still valuable for energetic purposes. This fuel could be used in current installations designed for traditional fuel. A comparison of the combustion process of low emission fuels and the combustion of traditional bituminous (black) coal showed that the emission of air pollution can be reduced with the use of smokeless fuels. Research has revealed that the emission of CO was lower than 30% v/v, benzo(a)pyrene was lower than 90% v/v, volatile organic compound (VOC) was lower than 80% v/v, and the particulates matter was reduced up to 90% when the traditional fuel was substituted with smokeless fuel [3]. In addition, for smokeless fuel combustion, a reduction of polycyclic aromatic hydrocarbons (PAH) and NO_x_ was observed when compared with traditional fuels, such as wood and coal combustion [4]. The smokeless char is characterized by the absence of tar and soot after the pyrolysis process. This keeps the exhaust channels and elements of the installation cleaner than in typical processes and provides a more uniform combustion process that makes airflow regulation and ember carrying easier. After thermal processing, the calorific value of smokeless coal was higher than traditional black coal or wood, and amounted to approximately 28 MJ/kg and was characterized by higher carbon content and lower volatile volume fraction when combusted [5].

The amount of particle board, OSB (oriented strand board), MDF/HDF (medium density fibreboard/high density fibreboard), and similar board types in production in 2018 were estimated to amount to 74 million m^3^ in EU and 15.7 million m^3^ in Poland [6]. The production of non-hazardous wood wastes in 2016 was estimated to be 53 million tons in EU and 2.6 million tons in Poland [7]. The amount of furniture waste in the EU was estimated at about 10 million tons annually [8]. The majority of furniture waste is based on wood composite boards, such as chipboard, plywood, medium-density fiberboard (MDF), and oriented strand board. These are wood-waste products prepared using wood chips, sawmill shavings, or sawdust pressed into sheets and hardened together with constituents like glue, synthetic resins, and other chemicals used to change their waterproof and fire-resistant properties. The amount of additional non-wood components is approximately 10% of the mass of the whole product. The presence of the listed additives in wooden products change the category of those materials and cannot be classified as a biomass according to EU directives (2010/75/UE). It is worth mentioning that wood products have interesting properties for energetic use, like high calorific values (approximately 17 MJ/kg [9]). There have been several applications proposed for wooden waste products’ usage, such as activated carbon production [10], briquette production for energetic purposes [11,12], adding wooden furniture waste in the process of fired clay brick production for the improvement of thermal insulation [13], and wood-polymer composite (WPC) production [14].

Pyrolysis of sawdust used as a smokeless fuel production was mentioned over six decades ago by Stamm and Harris [15]. Chembukulam et al. [16] presented their results on smokeless fuel preparation from the sawdust of several wood types. The authors observed that the optimum values of fuel characteristics might be obtained for the pyrolysis process at 538 °C. Ściążko et al. [17] described a series of analyses conducted to determine the quality of smokeless fuel. The experimental results proved the differences in terms of heat generation. The amount of traditional coal needed for the combustion process was 1.36 kg versus 1 kg of coal-based smokeless fuel. The authors described that comparison the combustion process of smokeless fuel compared with coal. Smokeless fuel was characterized by lower pollutant emission (e.g., approximately 50% lower CO emission), tar generation (approximately 5% of the amount produced by black coal), and dust emission (approximately 7% of dust produced with raw coal combustion). Arayici [18] used low-quality lignite as a fuel, which caused high smoke emissions in the combustion process due to its high tar content for smokeless fuel production. A two-step pretreatment process was conducted at 120 °C and pyrolysis at 450 °C with a heating rate of 150 °C/h. The product obtained was characterized with a tar content below 2%, the volatile matter was composed mostly of CH_4_ and H_2,_ and the calorific value was 23.6 MJ/kg. Numerous studies have focused on coal carbonization and briquetting for smokeless fuel production [19,20,21,22,23].

Low-rank coal and biomass (sawdust and olive stones) mixtures with different fractions were pyrolyzed by research groups for numerous studies [24,25,26,27,28] in order to produce high-quality smokeless fuel. Blesa et al. [24] observed a lower sulfur content and higher calorific values ranging from 26.5 MJ/kg to 32.1 MJ/kg, depending on the mixture in the prepared smokeless fuels. Other types of biomass, such as straw and almond shells [29], were used for smokeless fuel production, all with effects of carbon content and calorific value rises from 18–20 MJ/kg to 27–34 MJ/kg. Waste tires were used in the production of two types of bituminous coal using the slow pyrolysis process with a heating rate of 5 °C/min up to 900 °C [30]. The authors obtained degassed fuel (0.7–0.9%) characterized by a calorific value of approximately 33–34 MJ/kg, a total sulfur content of below 1.2 wt.%, and degassed (0.7–0.9%) [30]. Other types of wastes used by researchers for smokeless fuel production were wet, spent coffee grounds [31] waste risk husks [32], plastic waste materials [33], poplar chip [34], maize residues [35], tires [36], and even wet cake waste from the ethanol industry [37].

The aim of this study was to assess the possibility of using furniture waste for smokeless fuel production using the pyrolysis process. The novelty of this paper is, to the best of the authors’ knowledge, there are no other studies proposing furniture waste for smokeless fuel production. Four types of wood-based wastes were used in the pyrolysis process. Additionally, the slow and fast types of pyrolysis were compared, where the heating rates were 15 °C/min and 100 °C/min, respectively. The raw materials and the pyrolysis product yields were analyzed and compared. Activation of char has another possible use as an adsorbent [10], for applications in building materials [38], or as catalyst support for biomass conversion [39,40,41], and such studies have been planned for future. 

## 2. Materials and Methods

### 2.1. Materials

In this work, the waste from the furniture industry was used in the pyrolysis process. Four types of post-production wastes were chosen as raw materials and bought in the Polish building market. These were pine sawdust (PS), chipboard (CB), medium-density fiberboard (MDF), and oriented strand board (OSB). The pine sawdust, as a reference material, was a 100% biomass sample used as a raw material in furniture production. The CB sample contained raw wood material (spruce, pine), about 80–90%, urea-formaldehyde adhesive resin, hydrophobic agents, and decorative paper. The CB was flat pressed, three-layered board for internal use, which had been fragmented. The MDF sample consisted of about 80–90% raw wood materials (fir, pine), about 10% amine glue, and additives such as hardener and emulsion. The OSB sample consisted of up to 90% wood (pine) and resin binder urea-formaldehyde or polyurethane adhesive. The OSB was a flat-pressed three-layer board, which consisted of rectangular flat chips, which under high pressure and temperature, using synthetic resins as a binder, were pressed by hot rolling. All used furniture products were manufactured (in accordance with the manufacturer’s assurance) without the addition of halogenated compounds and wood protection agents. The pine sawdust was ground to pine strips with a diameter of 1–2 mm. The particle size of the CB, MDF, and OSB samples was about 2 × 4 cm. In Table 1, the pictures of the raw materials and char are presented.

### 2.2. Pyrolysis Process in Laboratory and in Microgram Scale

**Figure 1:**
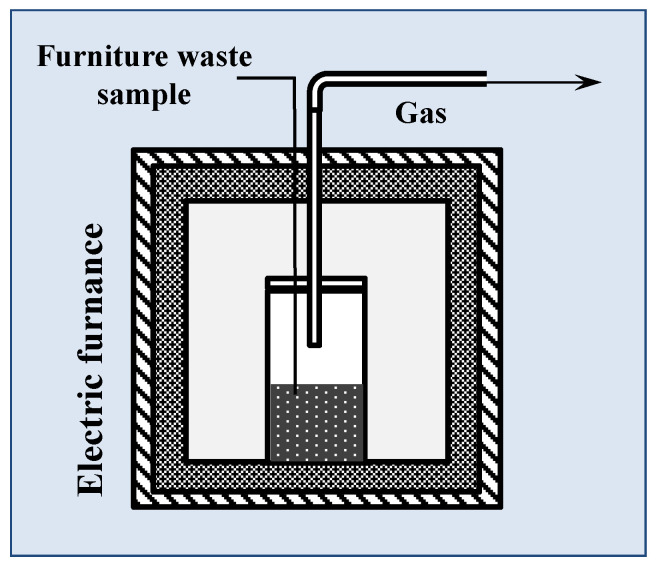
Schematic diagram of the pyrolysis laboratory-scale reactor [10].

The pyrolysis process of raw materials was conducted in a batch reactor located inside a laboratory-scale furnace (Neoterm MidiSUN lift 3.0 with thermoregulatory KXP3, Wrocław, Poland) (Figure 1). During the process, a steel reactor volume of 100 mL was put into the electric muffle furnace with the mass of sample about 10 g. The slow (SP) and fast (FP) types of pyrolysis were compared, where the heating rates were 15 °C/min and 100 °C/min, respectively. In SP, the sample was heated in the steel reactor from 25 to 600 °C. In fast pyrolysis, the steel reactor was put into a hot reactor (600 °C). The temperature of the sample was measured using a thermocouple. After each experiment, the reactors, with the char inside, were removed from the high-temperature furnace and, after cooling, were subjected to weighing and further testing. The microgram scale pyrolysis in a thermogravimetric analyzer (TG, 209 F3 Tarsus, Selb, Germany) was also used for comparison of the results. The experiments were conducted in a nitrogen atmosphere. 5 mg of samples were used and the heating rate was 15 °C/min, which corresponded to the slow pyrolysis process.

### 2.3. Characterization of Samples 

The elemental analysis and ash content of samples were analysed and used for calorific value calculations. The calorific value of the samples was calculated using equation [42]:

HHV = 349.4C + 1178H + 15.1N + 100.5S − 103.4O − 21.1 A

where, HHV represents high heating value; and C, H, N, S, O, and A represent carbon, hydrogen, oxygen, nitrogen, sulfur, and the ash content of material, respectively.

The elemental analysis of raw materials was conducted using a CHNS-O analyzer Flash 2000 (Thermo Scientific, Waltham, MA, USA). The solid products of the pyrolysis were analyzed using normal standardized methods. Moisture content was determined by a MAC moisture analyzer using CEN/TS 15414-1:2010; PN-EN 15414-3:2011 (Radwag, Radom, Poland); ash (PN-EN 15403:2011).

## 3. Results and Discussion

**Figure 2:**
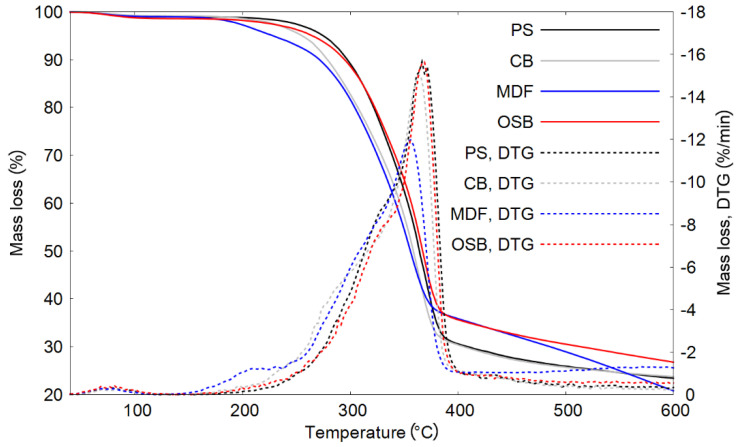
Thermogravimetric analysis and derivatives of mass loss of selected biomass samples.

The thermogravimetric analysis (TG) results presented an overall similarity of the pyrolysis process for all samples. The TG analysis was conducted for four samples of furniture waste, where the heating rate was 15 °C/min, which corresponded to the slow pyrolysis process used in this research. Analyzing the results, it can be observed (Figure 2) that within all of the other used samples, the MDF material started to decompose first at the lowest temperature (5% of mass loss at 226 °C). The most resistant to temperature treatment was the pine sawdust sample, which only lost 5% mass at 274 °C. Considering the progress of pyrolysis, it can be concluded that the mass-loss rate is a function of temperature for sawdust and OSB, and they are characterized by a similar slope. However, the chipboard and MDF were characterized with similar mass loss courses for temperatures below 350 °C. The slope and shape of the mass-loss curves stem from the composition of each of the materials. The board type samples, such as chipboard, MDF, and OSB, consist mainly of raw wood. Chipboard and MDF contain additional components (e.g., resin, glue) that decomposes at lower temperatures. At a final temperature of 600 °C, the solid residue ranged from 20.8% for MDF to 26.7% for OSB.

In Figure 2, the TG analysis results are presented as the rate of mass loss in %/min as a function of temperature. The summary of the TGA results is also presented in Table 2. The highest maximum mass-loss rate was observed for PS and it reached 1%/°C at 365 °C. Similar values were observed for CB (0.94%/°C at 365 °C) and OSB (0.97%/°C at 369 °C). The lowest maximum mass-loss rate was observed for MDF, which amounted to 0.7%/°C at 356 °C. This result is due to the fact that the decomposition process for MDF is more spread out over time than the decomposition of the other materials. The TG results are characteristic for samples of heterogeneous composition.

The pyrolysis process results in the decomposition of the biomass samples and obtained products into three fractions: solid, liquid, and gas. The solid fraction is characterized by a high-calorific value and high carbon content. The chemical energy of the biomass after the pyrolysis process is a result of the amount of obtained biochar and its calorific value. The results of solid fraction yield for slow and fast pyrolysis for the investigated samples are presented in Table 3. The weight of char ranged from 19.6% for the initial biomass mass for fast pyrolysis of PS to 31.8% for slow pyrolysis (SP) of CB. In general, higher char masses were obtained for SP and amount to a mean value of 32.5% of the initial biomass mass. For fast pyrolysis, the mean value was 23.1% of the initial mass. This is due to the generation of volatile matter in the pyrolysis process. In fast pyrolysis, particles of the solid fraction could be present in the gaseous fraction. The results of the two pyrolysis types are presented in Table 3.

The elemental components changed after the pyrolysis process. The amount of carbon and nitrogen rose, and the amount of hydrogen, oxygen, and ash decreased. The results of the elemental analysis for the biomass and biochars are presented in Table 4. In general, the carbon content rose from a range of 47–49% to 74–77% due to slow pyrolysis and to 80–86% due to fast pyrolysis. Compared with the raw fuel, the carbon content in the chars rose to 27.2% for SP and 34.2% for FP. The highest carbon content was observed for PS biochar after fast pyrolysis, which is a result of low ash content in the raw fuel. The increase in the carbon content is the main factor influencing the improvement of the heat value of the chars in relation to the raw fuel.

The hydrogen content decreased, which was a result of the degradation of the hydrocarbons (PAH) and the generation of methane and ethane in pyrolytic gas. As a result of these reactions, the hydrogen content decreased from 5.9% for raw material to 4.1% in char after slow pyrolysis and 2.9% in the char after fast pyrolysis. The decrease in hydrogen content had a negative effect on the calorific value. It was, however, compensated by an increase of carbon content and as a result, the final calorific value after pyrolysis was higher. The oxygen content was approximately 43% for the raw material. The obtained chars had no oxygen content that could react during the combustion process and resulted in a decrease in the higher heating value (HHV). After the pyrolysis process, the oxygen content in the char samples was bound to the metal oxides. In the combustion process, the oxide was in ashes. A decrease in the oxygen content was a result of the formation of carbon monoxide, carbon dioxide, and water. Carbon monoxide and dioxide formations are some of the first reactions during the pyrolysis process, and these two gasses are generated at low temperatures in the initial part of the process. 

**Figure 3:**
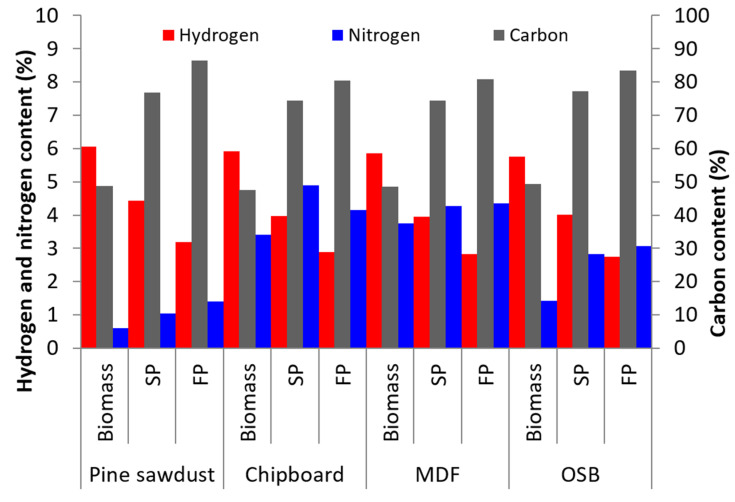
Elementary compounds in biomass and charcoals after pyrolysis.

Considering the results of the pyrolysis process (Figure 3), the raw material with a calorific value of about 17.3 MJ/kg turned into a char of HHV approximately 83.5% (approx. 29 MJ/kg) higher due to slow pyrolysis and 87.4% (approx. 31 MJ/kg) due to fast pyrolysis (Table 5). The pyrolysis process produced a product of a high calorific value, ranging from 28.4 MJ/kg for MDF to 29.9 MJ/kg for PS after slow pyrolysis and from 29.9 MJ/kg for CB to 33.1 MJ/kg for pine sawdust due to fast pyrolysis. Additionally, the difference between the char HHV values obtained after fast and slow pyrolysis was the highest for PS and amounted to an 8% difference between the two chars. 

**Figure 4:**
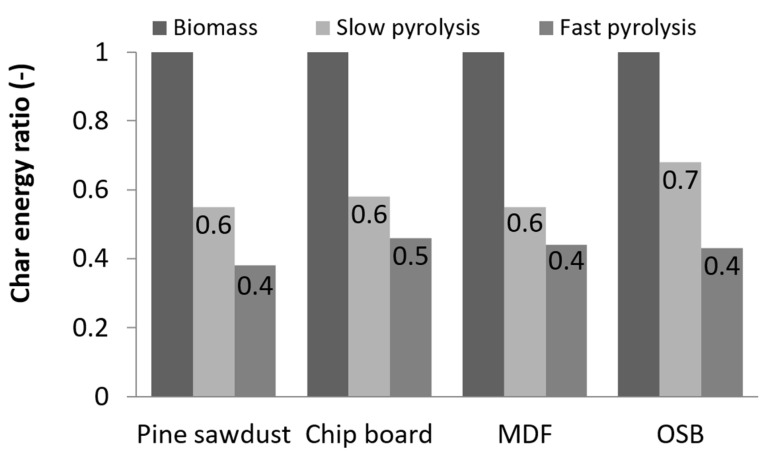
Energy loss as a result of the pyrolysis process.

Despite the fact that the char obtained in fast pyrolysis process was characterized with the highest calorific value, the obtained efficiency was low compared to the other investigated fuel types. Considering the calorific value and the amount of char, it can be observed that the slow pyrolysis process was more efficient in fuel generation. Comparing the results of the pyrolysis process in energetic terms, the energy (%) obtained from 1 kg of the raw material was calculated and compared to the energy in the initial raw material sample, as presented in Figure 4. The energy concentrated in the char after slow pyrolysis amounted to 55% for PS and up to 68% for OSB. In the case of fast pyrolysis, these values ranged from 38% for PS and up to 45% for CB. Analyzing these results, it is worth mentioning that the energy concentrated in char, calculated as the multiplication of HHV and solid pyrolysis product mass, was 27% higher for the slow pyrolysis process. Although the char after fast pyrolysis was characterized by a higher value of the heat of combustion, the efficiency of the process was lower and less char was obtained. The lowest energy value from 1 kg of biomass was held by PS char and obtained with fast pyrolysis, despite the fact that it had the highest HHV among all of the other samples. 

**Figure 5:**
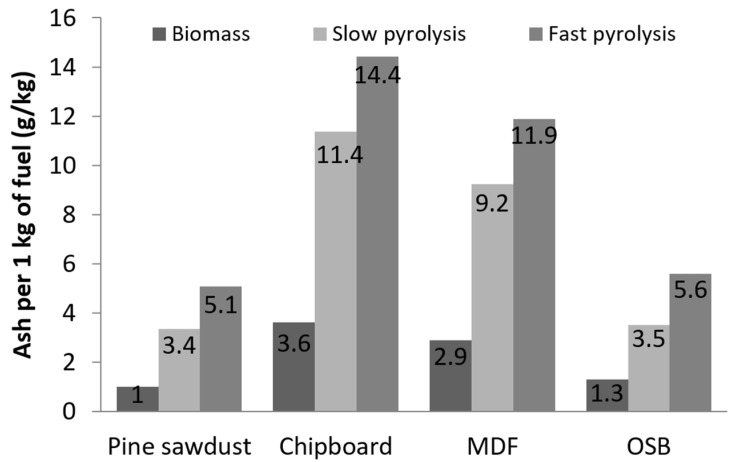
Ash mass per 1 kg of biomass and biochar mass.

The pine sawdust sample was characterized by the lowest energy value obtained during fast pyrolysis from 1 kg, despite the fact that the PS biochar had the highest HHV. Considering the use of char as a fuel in the combustion process, the ash content is an important parameter. In a comparison of both processes, combustion and pyrolysis, the mineral fraction, such as metals oxides, additives and others, is part of char in pyrolysis and ash in combustion. The ash content analysis for the investigated materials is presented in Figure 5. The ash content in the raw material was approximately 0.9 g for PS and up to 3.6 for CB. This significant increase was observed for all of the char samples, and the value ranged from 3.3 g for PS up to 11.4 g for CB for slow pyrolysis and 5.1 g for PS up to 14.4 g for CB for fast pyrolysis. The ash content in the chars compared to raw fuel increased 3.1 times for slow pyrolysis and 4.4 times for fast pyrolysis.

**Figure 6:**
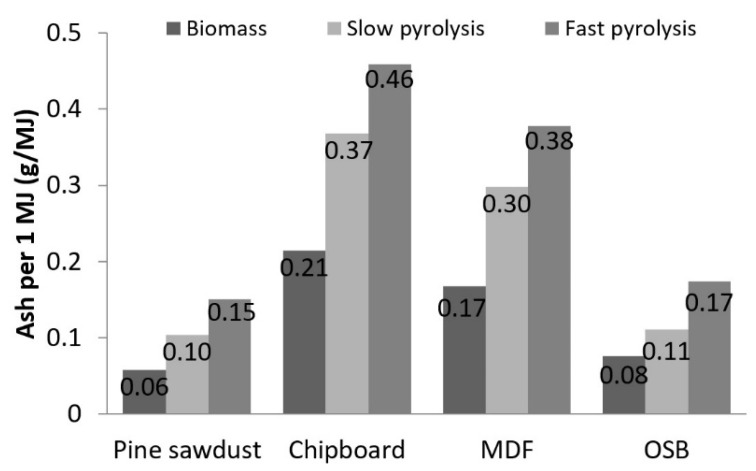
Ash content per 1 MJ of biomass and biochar energy.

The significant increase in the ash content per 1 kg of the raw fuel occurred at the same time the calorific value increased. Therefore, the increase of the ash content per 1 MJ of energy was not that high. The increase in the ash content per energy unit (Figure 6) was the most significant change in fast pyrolysis for PS (up to 160%). The lowest value, 45%, was observed for OSB after slow pyrolysis. The comparison of the ash content in the chars and the raw materials showed an increase on average of 1.7 times for slow pyrolysis and 2.3 times for fast pyrolysis.

## 4. Conclusions

Furniture waste is a promising material in terms of smokeless fuel production. The calorific values of the chars obtained from the pyrolysis process do not differ significantly from the char obtained from the raw wood (pine sawdust). The differences are insignificant, and all of the chars have an HHV value higher than 28 MJ/kg. Higher calorific values were obtained for chars produced with the fast pyrolysis process. Fast pyrolysis provided a lower amount of chars and consisted of higher ash content. In the slow pyrolysis process, the amount of the ash content was approx. 1.7 times higher (per MJ) and in the fast pyrolysis, it was approx. 2.3 times higher. This proves that slow pyrolysis was more efficient in terms of smokeless fuel production. The use of the pyrolysis process for smokeless fuel production is appropriate for furniture waste and not for the ‘clean’ wooden wastes. After the pyrolysis process, furniture waste kept higher chemical energy amounts in the chars when compared to the pyrolyzed wood. Moreover, the high ash content in the chars is compensated by their higher calorific values and its low volatile content makes the combustion process more ecological and less complicated. 

Smokeless fuel produced from wood wastes has one major advantage over raw wood wastes: namely, the exhaust gas produced during smokeless fuel combustion contains much less tar and solid pollutants. Therefore, its application for energy production provides less soot and contamination of the heat transfer surface and extends the life time of heating devices and heating appliances. 

Rough estimations based on the data presented in introduction (wood waste production) and the presented results (assuming an average solid fraction yield of 25%) show that the combustion of smokeless fuel would eliminate 2.3 million tons of CO_2_ emissions in Poland and 48.58 million tons of CO_2_ in EU. These calculations are carried out with assumption that all wood waste would decompose similarly to the presented results.

## Figures and Tables

**Table 1:** Raw materials before and after pyrolysis process.

Type	Pine Sawdust	Chipboard	Medium-Density Fiberboard	Oriented Strand Board
**Raw Material**	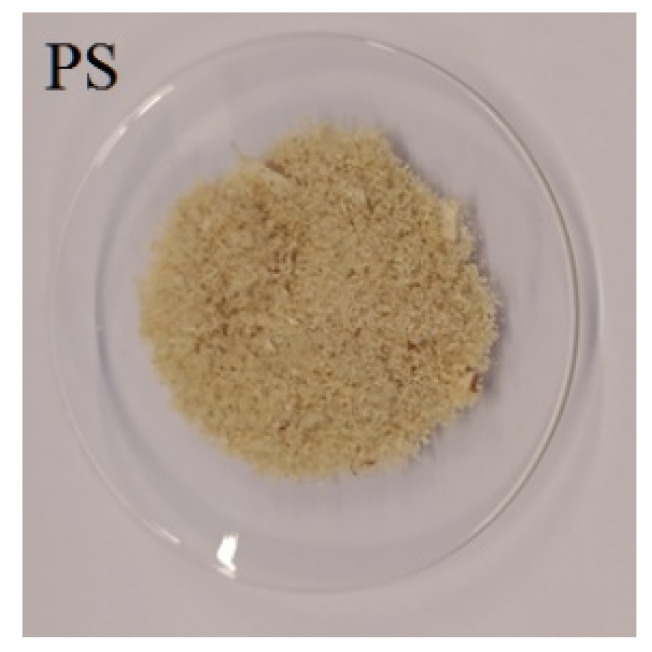	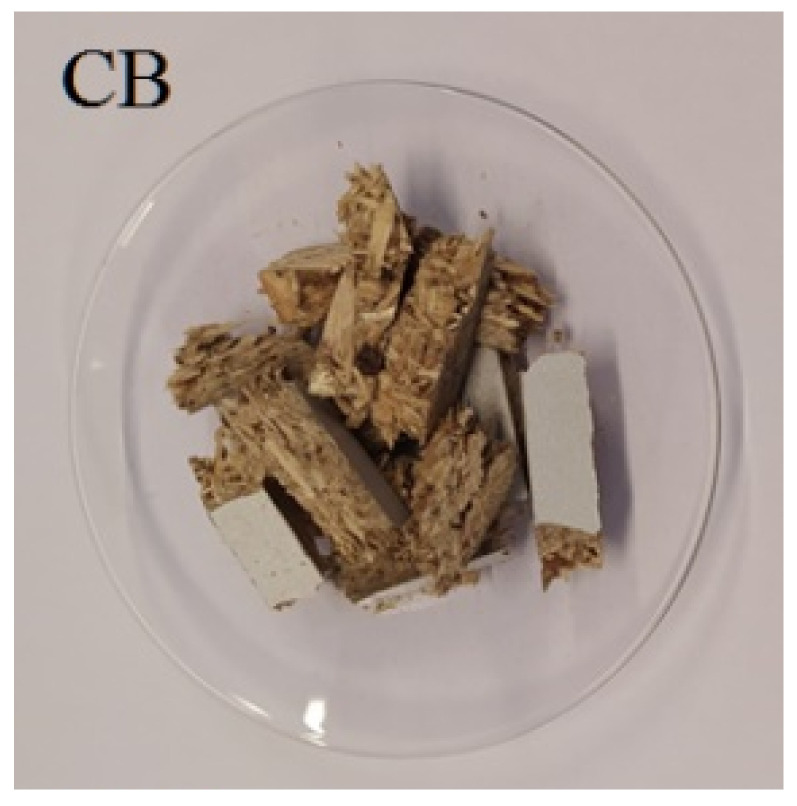	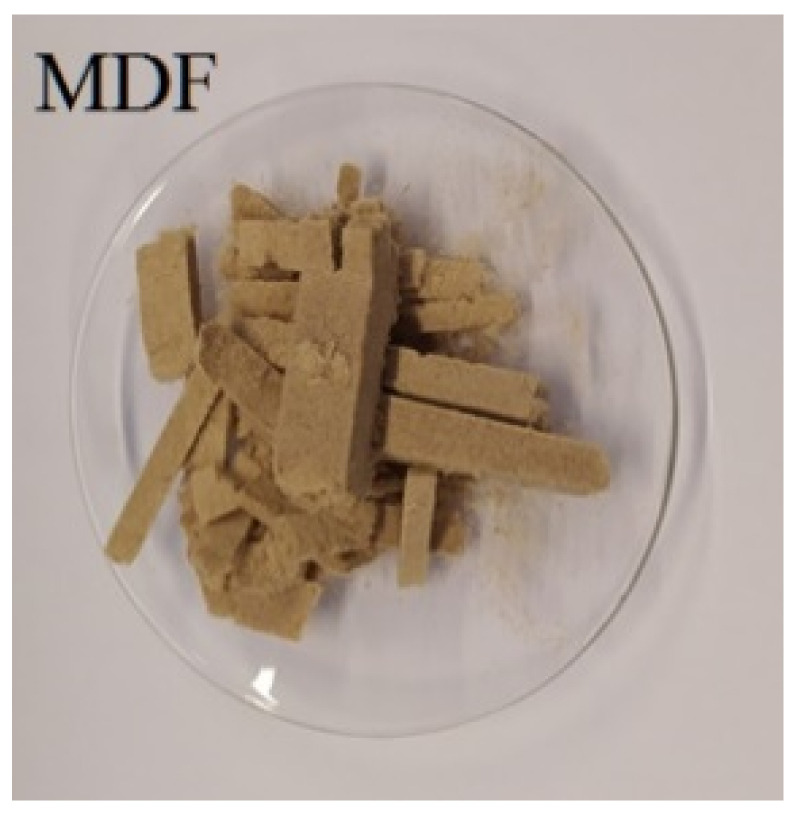	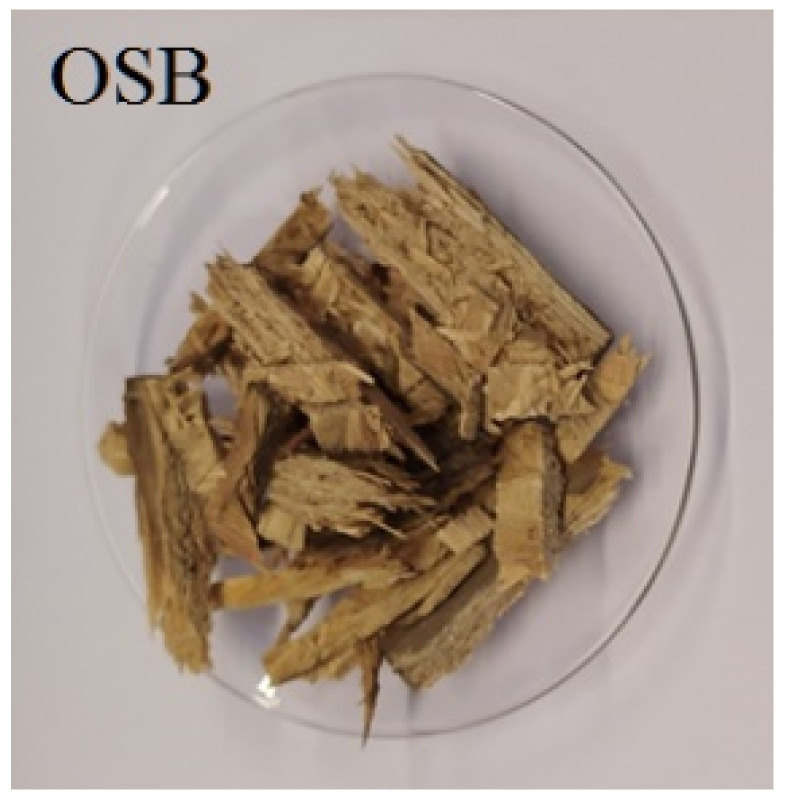
**Char**	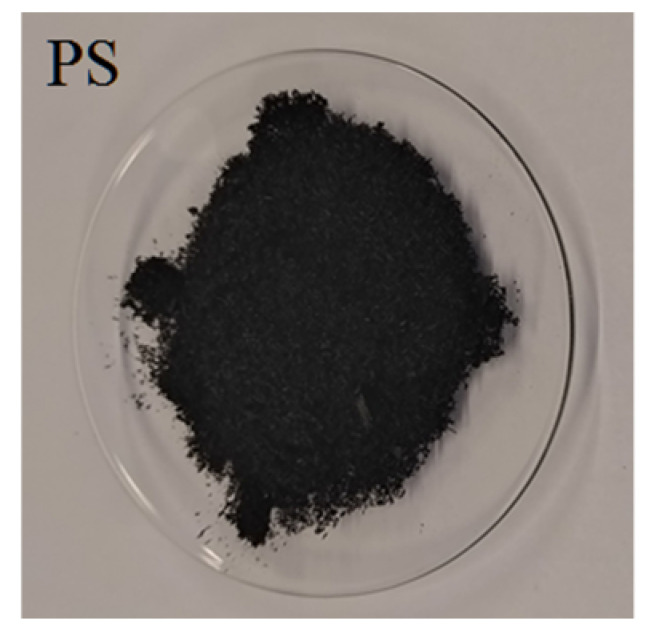	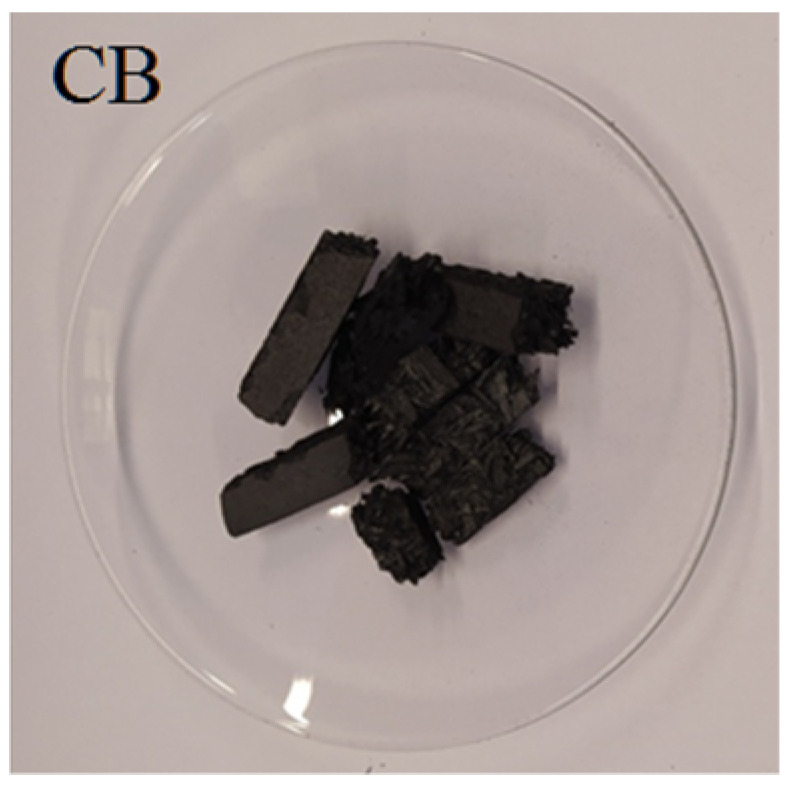	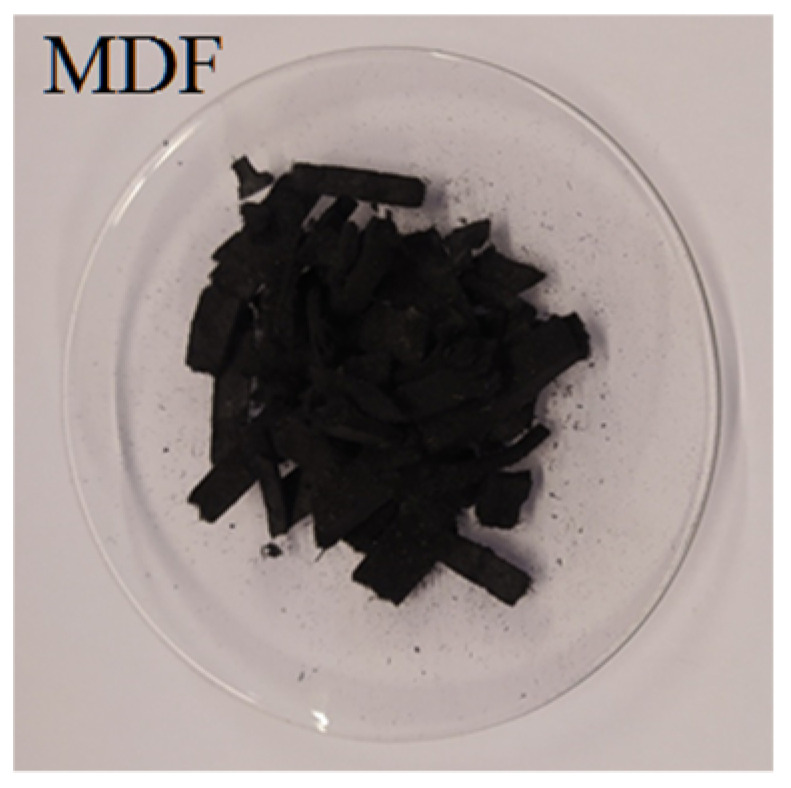	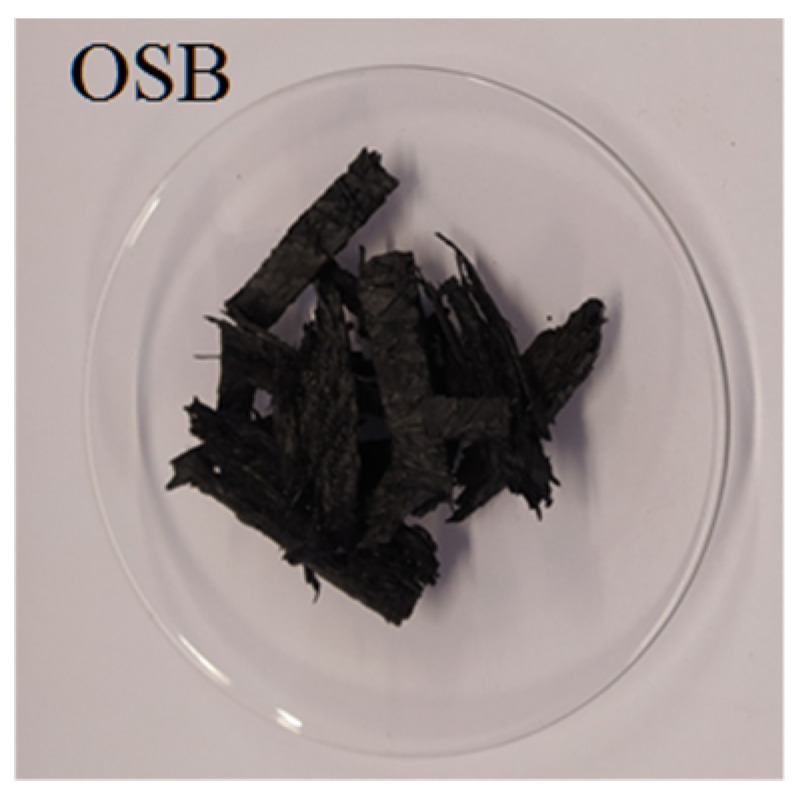

**Table 2:** Thermal decomposition characteristics of furniture waste samples pyrolyzed in a nitrogen atmosphere.

Raw Material	Decomposition Temperature (°C)			DTG Maxima (°C)		Solid Residue (%)
	T_5%_	T_10%_	T_50%_	T_max_	% of Mass/°C	T600
PS	274	297	365	367	1.00	23.4
CB	255	278	358	365	0.94	23.7
MDF	226	271	355	356	0.77	20.8
OSB	263	293	365	369	0.97	26.7

**Table 3:** Comparison of solid fraction yields from slow and fast pyrolysis.

Raw Materials	Slow Pyrolysis (15 °C/min)	Fast Pyrolysis (100 °C/min)
**PS**	29.6%	19.6%
**CB**	31.8%	25.1%
**MDF**	31.4%	24.4%
**OSB**	37.0%	23.3%

**Table 4:** Chemical composition of examined biomasses and respective biochars.

	Carbon Content (%)			Hydrogen Content (%)			Nitrogen Content (%)		
Biomass type	Biomass	Biochar (SP)	Biochar (FP)	Biomass	Biochar (SP)	Biochar (FP)	Biomass	Biochar (SP)	Biochar (FP)
PS	48.8	76.9	86.4	6.1	4.4	3.2	0.6	1.0	1.4
CB	47.5	74.5	80.4	5.9	4.0	2.9	3.4	4.9	4.2
MDF	48.5	74.5	80.8	5.9	4.0	2.8	3.8	4.3	4.4
OSB	49.4	77.2	83.5	5.8	4.0	2.8	1.4	2.8	3.1

**Table 5:** Calorific values of examined fuels and respective biochars.

HHV (MJ/kg)			
Biomass Type		Pyrolysis Type	
		SP	FP
	raw material	char	char
PS	17.41	29.90	33.14
CB	17.03	28.55	29.85
MDF	17.53	28.40	30.02
OSB	17.38	29.56	31.11
